# End malaria for good: a review of current strategies and future novelties for malaria elimination in Nigeria

**Published:** 2018-03-01

**Authors:** Omosivie Maduka

**Affiliations:** 1 Department of Preventive and Social Medicine, College of Health Sciences, University of Port Harcourt, Nigeria

## Abstract

Malaria is endemic in 91 countries and territories. Currently, over half of the world’s population is at risk for malaria with malaria prevalence in sub-Saharan Africa remaining the highest in the world. Nigeria accounts for 56% of malaria cases in the West African sub-region. Malaria control is historically the oldest control programme in Nigeria, having been in existence since 1948. Malaria control in Nigeria is guided by National Malaria Strategic Plans. The goal of the NMSP (2014-2020) is ‘to reduce malaria burden to pre-elimination levels and bring malaria-related mortality to zero’ using strategies under seven strategic objectives. The theme for the 2017 World Malaria Day activities was ‘End Malaria for Good’. This theme indicates a sustained push for national and international commitments toward goal zero. Although the prevalence of malaria has dropped significantly, from 42% in 2010 to 27.4% in 2015, a lot of effort needs to be made to actualise a malaria-free Nigeria. This review discusses the current strategies in place to control and eliminate malaria. It also describes some future novelties available to sub-Saharan Africa and Nigeria to ‘End Malaria for Good.’

## 1 Introduction

On the 25^th^ of April each year, we celebrate World Malaria Day (WMD). On this day we bring to the fore all the efforts and activities focused towards achieving the goals of malaria control, elimination and ultimately eradication. The theme for the 2017 WMD was ‘*End Malaria for Good.’* This theme is a rolled over theme from the 2016 WMD, indicating a sustained push for international commitments toward achieving goal zero. In the words of the former WHO Director-General Margaret Chan: *“Any goal short of eradicating malaria is accepting malaria; it’s making peace with malaria; it is rich countries saying: ‘We don’t need to eradicate malaria around the world as long as we’ve eliminated malaria in our own countries.’ That’s just unacceptable.”*

### 1.1 Malaria prevalence and incidence in the World

Malaria is currently endemic in 91 countries and territories, a reduction from 108 countries in 2000. Currently, over half of the world’s population is at risk for malaria with prevalence in sub-Saharan Africa remaining the highest in the world. Currently, 43 countries in sub-Saharan Africa are endemic for malaria. However, malaria prevalence rates have dropped in the sub-continent from between 20 to above 40% since 2000 [1].

The estimated global incidence rate of malaria decreased by 21% between 2010 and 2015 and 41% between 2000 and 2015. Also, the proportion of the population at risk in sub-Saharan Africa infected with malaria parasites is estimated to have declined from 22% in 2005 to 17% in 2010, and to 13% in 2015 [1,2]. The global technical strategy for malaria 2016-2030 describes an ambitious roadmap for a malaria-free world with huge achievements by 2030. By this date, we should have achieved a 90% reduction in malaria mortality rates and malaria incidence worldwide in comparison to the 2015 proportion of 13% [2]. We should also have eliminated malaria from an additional 35 countries and successfully prevented re-establishment of malaria into the countries which have successfully eliminated it [1].

The agreed targets for malaria eradication are achievable in line with the principles entrenched in the Sustainable Development Goals (SDG) agenda. The mainstreaming of SDGs themes such as encouraging financial and political commitment, country leadership, multi-sectoral partnership, technical knowledge, the involvement of civil society organisations and partnerships with academic and research institutions, into malaria control and elimination efforts have made the war against malaria a model SDG project [3].

### 1.2 Malaria control and elimination in Nigeria

Nigeria accounts for 56% of malaria cases in the West African sub-region. Microscopy detected malaria prevalence in Nigeria dropped from 42% in 2010 to 27.4% in 2015. However, great variations still exist among regions within the country. The 2015 malaria prevalence (among children 6 to 59 months) ranged from 37.1% in the northwest to 16.6% in the southwest with the highest prevalence of 63.6% in Kebbi (n=157) and lowest prevalence of 0% in Lagos (n=246). A zero prevalence in Lagos state, though noteworthy, does not necessarily indicate the absence of malaria parasites in the state but rather the absence of parasites in the 246 children aged 6 to 59 months sampled across the state [1,4].

Malaria control is historically the oldest control programme in Nigeria, having been in existence since 1948. It has gone through several transitions from the National Malaria Service to the National Malaria Control Programme in 1986, to the National Malaria Elimination Programme in 2013 as a reflection of the country’s desire for a malaria-free nation. The National Malaria Elimination Programme and its State Elimination Programmes are domiciled in the Ministries of Health and are tasked with oversight, regulatory and programme management functions relating to all malaria control activities in the country and its states [5]. The National Malaria Strategic Plans (NMSP) have, over the years, served as the blueprint of malaria control and elimination objectives and targets. Four NMSPs have been in use with the latest being the NMSP 2014-2020. The goal of this latest NMSP is ‘*to reduce malaria burden to pre-elimination levels and bring malaria-related mortality to zero’* through activities under seven strategic objectives [6].

### 1.3 NMSP strategic objectives 2014-2020

1. At least 80% of targeted populations utilise appropriate malaria preventive measures by 2020;
2. To ensure that all persons with suspected malaria who seek care are tested with RDT or microscopy by 2020;
3. All persons with confirmed malaria seen in private or public health facilities receive prompt treatment with an effective anti-malarial drug by 2020;
4. At least 80% of the population practice appropriate malaria prevention and management by 2020;
5. To ensure the timely availability of appropriate antimalarial medicines and commodities required for prevention, diagnosis, and treatment of malaria in Nigeria by 2018;
6. All health facilities report on key malaria indicators routinely by 2020;
7. To strengthen governance and coordination of all stakeholders for effective programme implementation towards an ‘A’ rating by 2020 on a standardised scorecard.

## 2 Malaria interventions in Nigeria: situation analysis, challenges and recommendations

### 2.1 Malaria prevention

The NMSP 2014-2020 listed five key intervention areas for malaria prevention activities. These include: universal access to Long-Lasting Insecticide-treated nets (LLINs), indoor residual spraying (IRS), larval source Management (LSM), provision of Intermittent Preventive Treatment of malaria in pregnancy (IPTp) to all pregnant women attending antenatal clinics in targeted districts, vector sentinel surveillance and resistance monitoring and quality assurance of commodities.

### 2.2 Long-Lasting Insecticide Treated Nets (LLINs)

LLINs have been lauded as the mainstay of malaria prevention, especially in sub-Saharan Africa [1,2]. As of 2015 net ownership across the Nigeria was 69% [4]. This is a steep increase from ownership of 2% in 2003. Over 103.8 million LLINs were distributed in Nigeria between 2009 and 2015 with higher net ownership in rural compared to urban populations [4,5]. Across the regions in the country the northwest has the highest LLIN ownership while southwest has the lowest.

Net utilisation is yet to come to par with net ownership. There are persistently lower rates for net utilisation compared to ownership. However, net utilisation rates (measured as the proportion of persons who slept inside a treated net the previous night) has risen over the past decade. Among children, less than five years old, net utilisation has gone up from 1% in 2003 to 39% in 2015 while among pregnant women, utilisation increased from 5% in 2003 to 43% in 2015 [4,5].

The challenges mitigating against increased net ownership and utilisation are manifold. In spite of all the social mobilisation campaigns around net ownership and use, many Nigerians still find sleeping under a treated net unacceptable. Socioeconomic and sociocultural barriers such as irregular power supply, the perception that net use is for women and children, perceptions surrounding itching, colour, odour, and heat production, among others, impede net use. There are also concerns for sustainability since virtually all the net hanging campaigns have been capital intensive and donor driven. There is currently only one indigenous firm manufacturing LLINs in the country. There is need to support indigenous LLIN manufacturers, forge sustainable partnerships with civil society groups and community-based organisations for low budget net distribution, hanging and monitoring campaigns, and partner with mobile and web-based health tools to create demand for LLIN use.

### 2.3 Indoor Residual Spraying (IRS)

IRS is also a very effective intervention for rapid reduction of malaria transmission [2]. In 2015, 106 million persons around the globe were protected with IRS. IRS is used only in particular areas. The proportion of the population at risk protected by IRS declined from a peak of 5.7% globally in 2010 to 3.1% in 2015, and from 10.5% to 5.7% in sub-Saharan Africa [2]. Specifically, in Nigeria, the 2015 Malaria Indicator Survey (MIS) reported that only 1% of households surveyed in the country had received IRS within the preceding 12 months [4]. Reasons for this within the Nigerian context may relate to the prohibitive cost of IRS campaigns, the absence of vector maps to guide implementation and the rising incidence of resistance to pyrethroids and other insecticides. There is a need, therefore, to invest in vector mapping and insecticide resistance monitoring.

### 2.4 Prevention among vulnerable populations

Prevention among vulnerable populations involves providing Intermittent Preventive Treatment for pregnant women (IPTp) and infants (IPTi) and providing seasonal chemoprevention (SMC) for children less than five years of age. IPTp entails giving three or more doses of sulphadoxine-pyrimethamine (SP) as directly observed treatment (DOTS), one month apart from after the onset of quickening. The uptake of the three-dose IPTp in Nigeria is low at 19% in 2015 [4]. However, uptake of two-dose IPTp was 37% [4]. Both figures reflect poor implementation of IPTp and poor knowledge and acceptance of three or more doses of IPTp. Although the National Malaria Policy recognises the role of IPTi and SMC, implementation is still poor. SMC has been largely untapped as a viable option for prevention of transmission in the Sahel regions of the country especially as parasite prevalence rates continue to drop. This is one intervention that has huge prospects for getting us to the goal of zero deaths from malaria by 2020.

### 2.5 Supplementary vector control

Supplementary vector control methods include larval source management (LSM) and personal protection. LSM has been advocated as useful for the control of breeding sites only where larval breeding sites are few, fixed and findable. Although LSM has been implemented in Rivers and Lagos states, it hasn’t had much impact probably because of operational, technical, and logistical challenges associated with this vector control approach.

### 2.6 Diagnosis and treatment

The National Malaria Policy states that *‘All suspected cases of malaria should have a parasite-based confirmation before the institution of antimalarial treatment at all levels of healthcare delivery in the country; except in extraordinary circumstances where the diagnostic facility is not available.’*

This is an emphatic statement about the country’s thrust towards ensuring that all persons with suspected malaria who seek care are tested with RDT or microscopy by 2020. This completely rules out presumptive diagnosis as an acceptable strategy. As a result of this, the rates of testing with RDT and microscopy within health facilities has increased significantly. Studies show that 70 to 90% of persons reporting fever in health facilities (public or private) receive a diagnostic test for malaria [5]. The role of mRDTs in malaria control efforts has been well documented [7–9]. RDTs for malaria began to be available on a large scale in Nigeria in 2010 [10,11]. While the recognition of RDTs as a reliable and cost-effective test for parasite-based diagnosis of malaria has grown, it has encountered challenges [7,9,12]. The ability of mRDTs to detect low levels of parasitaemia has often been challenged. Also, there is some element of subjectivity involved in reading the results, leading in some situations to the rejection of mRDT results and treatment of patients with malaria in the presence of mRDT negative results [6,10,11,13]. Innovations such as the ‘Deki reader’ improve the accuracy of mRDTs as well as provide a platform for real-time data capture. Microscopy remains the diagnostic gold standard. Unfortunately, in Nigeria, light microscopy is still plagued with many challenges relating to the technical skill of microscopists, availability of electricity, consumables, and logistics to provide valid malaria test results.

The NMSP strategic objective for malaria treatment is to ‘*ensure that all persons with confirmed malaria seen in private or public health facilities receive prompt treatment with an effective anti-malarial drug by 2020*’. Effective treatment as defined by the National Malaria Policy refers to the use of Artemisinin-based combination therapy (ACT) specifically Artemether-Lumefantrine or Artesunate-Amodiaquine combinations. ACT availability within the country stands at 97% [14]. The facilitators for this include the partnerships for affordable medicine facilities that drove down prices of ACTs and ensure availability at all levels of care. However, persistent and widespread availability and use of chloroquine and other monotherapies raise cause for concern [5].

## 3 Future novelties

Over the next decade or two, greater gains are likely to be won against malaria. These gains will most likely be fostered by game-changing novelties that improve our ability to prevent and interrupt transmission and to track, test and treat malaria. Innovations that improve the ability to detect malaria parasite such as urine testing for *Plasmodium* species is already within reach in the country and needing mass production and distribution at affordable cost [15]. This will make home/self-testing for malaria feasible. Improving the sensitivity and specificity of current mRDTs through the use of automated RDT readers such as the ‘Deki-reader’ would also be a step in the right direction [16].

Information and communication technologies for implementing web and mobile-based technologies for vector and parasite surveillance, geo-mapping and statistical modelling will also play a key role in the war against malaria. Technologies that help improve the uptake and use of LLINs through phone and social media reminder and support systems are being piloted. Research and development for new chemicals that halt insecticide resistance in its tracks and better and more effective drug treatments are ongoing. Stakeholders in phytomedicine need to be on the cutting edge of new formulations that can be a viable replacement for ACTs.

Finally, more than 20 malaria vaccine candidates are in various stages of development; of these, RTS, S/AS01 (known as "RTS, S") is the most advanced. The vaccine has been shown in clinical trials to provide partial protection against *P. falciparum* malaria in young children [2]. On April 24, 2017, the WHO Regional Office for Africa (WHO/AFRO) announced a pilot implementation programme beginning in 2018. The RTS, S vaccine will be available in three African countries—Ghana, Kenya, and Malawi. These countries were chosen for having mature existing immunisation programmes and high coverage of insecticide-treated nets, yet with persistently high malaria burdens [17].

## 4 Conclusions

The WHO Global Technical Strategy is insistent on eliminating malaria from an additional 35 countries by the end of 2030. This is barely 12 years away. Will Nigeria join other countries in making history? The answer to this question lies in our ability to provide homegrown and lasting solutions to the challenges facing scale up and consistent implementation of tried and tested malaria interventions. Particular focus needs to be made to the economic and social development as the milieu for effective implementation of the National Malaria Policy. We also need to pay attention to and support homegrown technologies and innovations in prevention, diagnosis, treatment and other supportive/cross-cutting activities. We need to encourage sustainable implementation of malaria interventions within the context of a robust and resilient health system, health care financing, poverty alleviation, multi-sectoral partnerships and good governance.
